# Prevalence of and risk factors for iron deficiency among pregnant women with moderate or severe anaemia in Nigeria: a cross-sectional study

**DOI:** 10.1186/s12884-023-06169-1

**Published:** 2024-01-05

**Authors:** Ochuwa Adiketu Babah, Opeyemi Rebecca Akinajo, Lenka Beňová, Claudia Hanson, Ajibola Ibraheem Abioye, Victoria Olawunmi Adaramoye, Titilope A. Adeyemo, Mobolanle Rasheedat Balogun, Aduragbemi Banke-Thomas, Hadiza S. Galadanci, Nadia A. Sam-Agudu, Bosede Bukola Afolabi, Elin C. Larsson

**Affiliations:** 1 Department of Global Public Health, Karolinska Institutet, Stockholm, Sweden; 2 Faculty of Clinical Sciences, College of Medicine, University of Lagos, Idi-Araba, Lagos, Nigeria; 3 Department of Obstetrics and Gynaecology, Lagos University Teaching Hospital, Idi-Araba, Lagos, Nigeria; 4 Centre for Clinical Trials and Implementation Science (CCTRIS), College of Medicine, University of Lagos, Idi-Araba, Lagos, Nigeria; 5 Department of Public Health, Institute of Tropical Medicine, Antwerp, Belgium; 6 Department of Global Health and Population, Harvard T.H. Chan School of Public Health, Boston, MA USA; 7 Department of Haematology and Blood Transfusion, Lagos University Teaching Hospital, Idi-Araba, Lagos, Nigeria; 8 Department of Community Health, Lagos University Teaching Hospital, Idi-Araba, Lagos, Nigeria; 9 Global Maternal and Newborn Health Hub, Institute of Lifecourse Development, University of Greenwich, London, UK; 10 African Center of Excellence for Population Health and Policy, Bayero University, Kano, Nigeria; 11 Department of Obstetrics and Gynaecology, College of Health Sciences Bayero University Kano/ Aminu Kano Teaching Hospital, Kano, Nigeria; 12 International Research Center of Excellence, Institute of Human Virology Nigeria, Abuja, Nigeria; 13 Institute of Human Virology, University of Maryland School of Medicine, Baltimore, USA

**Keywords:** Anaemia, Iron deficiency, Pregnancy, Prevalence, Risk factors, Diet, Food frequency, Nigeria

## Abstract

**Background:**

Anaemia during pregnancy causes adverse outcomes to the woman and the foetus, including anaemic heart failure, prematurity, and intrauterine growth restriction. Iron deficiency anaemia (IDA) is the leading cause of anaemia and oral iron supplementation during pregnancy is widely recommended. However, little focus is directed to dietary intake. This study estimates the contribution of IDA among pregnant women and examines its risk factors (including dietary) in those with moderate or severe IDA in Lagos and Kano states, Nigeria.

**Methods:**

In this cross-sectional study, 11,582 women were screened for anaemia at 20-32 weeks gestation. The 872 who had moderate or severe anaemia (haemoglobin concentration < 10 g/dL) were included in this study. Iron deficiency was defined as serum ferritin level < 30 ng/mL. We described the sociodemographic and obstetric characteristics of the sample and their self-report of consumption of common food items. We conducted bivariate and multivariable logistic regression analysis to identify risk factors associated with IDA.

**Results:**

Iron deficiency was observed among 41% (95%CI: 38 – 45) of women with moderate or severe anaemia and the prevalence increased with gestational age. The odds for IDA reduces from aOR: 0.36 (95%CI: 0.13 – 0.98) among pregnant women who consume green leafy vegetables every 2-3 weeks, to 0.26 (95%CI: 0.09 – 0.73) among daily consumers, compared to those who do not eat it.

Daily consumption of edible kaolin clay was associated with increased odds of having IDA compared to non-consumption, aOR 9.13 (95%CI: 3.27 – 25.48). Consumption of soybeans three to four times a week was associated with higher odds of IDA compared to non-consumption, aOR: 1.78 (95%CI: 1.12 – 2.82).

**Conclusion:**

About 4 in 10 women with moderate or severe anaemia during pregnancy had IDA. Our study provides evidence for the protective effect of green leafy vegetables against IDA while self-reported consumption of edible kaolin clay and soybeans appeared to increase the odds of having IDA during pregnancy. Health education on diet during pregnancy needs to be strengthened since this could potentially increase awareness and change behaviours that could reduce IDA among pregnant women with moderate or severe anaemia in Nigeria and other countries.

**Supplementary Information:**

The online version contains supplementary material available at 10.1186/s12884-023-06169-1.

## Introduction

Anaemia in pregnancy is a major public health challenge which caused 50 million disability-adjusted life years (DALYs) worldwide in 2019 [1]. Globally, it affected 500 million women of reproductive age 15 – 49 years in the same year; and of these, 32 million were pregnant women [1]. Anaemia is among the leading causes of maternal and perinatal morbidity and mortality globally [2, 3]. The burden of the condition is greater in low- and middle-income countries compared to high-income countries. The variation in anaemia prevalence by region is partly a reflection of the global inequalities in nutrition, literacy level and access to health care; all of which are poorer in low-middle-income countries [4–6].

The regions with the highest burden of anaemia in pregnancy are South-East Asia and sub-Saharan Africa, where it affects 244 million and 106 million women of reproductive age, respectively [1]. These two regions are the most populous in the world with a population of 4.8 billion in Asia and 1.5 billion in Africa, each contributing 59% and 18% respectively to the world’s population [7]. The high prevalence of anaemia in these regions is likely because many people are in the poorest wealth quintiles, with low level of educational attainment, and high burden of malnutrition in form of micronutrient deficiencies like iron, folate, zinc, and vitamin B12 deficiencies [8–11].

Anaemic pregnant women have higher odds for maternal complications like anaemic heart failure, preterm delivery, and postpartum haemorrhage; and a 3.5 times increased risk of death in those with severe anaemia [2, 12]. It is associated with a higher incidence of adverse perinatal outcomes like foetal growth restriction, foetal demise, babies with birth asphyxia, and neonatal death; 62% in anaemic compared to 28% in non-anaemic pregnant women [3, 13].

Serum ferritin is widely used in research and clinical settings for screening for IDA [14]. However, universal screening during pregnancy might be challenging in low-middle-income countries due to inadequate laboratory facility, low staff strength, high antenatal patient load, and poverty. Treatment for IDA in pregnancy includes oral and parenteral iron supplementation. Prevention and treatment also include dietary modifications to improve both the intake and the bioavailability of dietary iron [15, 16]. Poor compliance with oral iron treatment is a problem and is often because of suboptimal adherence associated with multiple dosing, side effects which are mostly gastrointestinal in nature, or poverty [17, 18]. Dietary factors such as inadequate intake of meat and green vegetables significantly increase the odds of having anaemia during pregnancy [19].

[20–24]. In Nigeria, the prevalence of anaemia among women of reproductive age 15 – 49 (defined as finger prick haemoglobin concentration less than 11 g/dL) was 58% based on the most recent nationally representative Nigeria Demographic and Health Survey (NDHS) 2018 [25]. The survey found prevalence of anaemia in pregnancy to be 50% in Lagos state and 47% in Kano state, Nigeria [25].

In particular, iron deficiency was reported in 25 – 46% of pregnant women with anaemia in Nigeria [26]. Iron is a vital component of haematopoiesis [27]. In a recent systematic review from Nigeria, multiparity, being in the third trimester of pregnancy, and low socioeconomic status were identified as risk factors for iron deficiency anaemia (IDA) among pregnant women [26]. In most health facilities in Nigeria routine screening for anaemia in pregnancy is determined by haematocrit, whereas specific screening for IDA is uncommon. As many as 17% of Nigerian women who registered for ANC do not receive iron supplementation; and of those who do, 17% will not take the iron supplements while 91% of those who will take the iron supplements have poor adherence with the dosage [28].

There is a paucity of studies assessing prevalence and risk factors for IDA in pregnancy in Nigeria; and none exploring dietary risk factors [26]. The only Nigerian study, found in the literature, on dietary intake and IDA, considered IDA to be same as anaemia in pregnancy and defined IDA as haemoglobin concentration < 11 g/dl [29]. Understanding the factors associated with IDA may help improve counselling of pregnant women, especially with respect to diet which is a modifiable factor.

This study focused on pregnant women with moderate or severe anaemia as this group of pregnant women will often need further evaluation and treatment because of their higher risk of suffering negative consequences during pregnancy [30]. It estimated the contribution of IDA among pregnant women and examined its risk factors (including dietary) in those with moderate or severe IDA in Lagos and Kano states, Nigeria.

## Methods

### Study setting

This cross-sectional study was conducted at 11 publicly owned health facilities (five at primary, four at secondary, and two at tertiary) in Lagos and Kano states, Nigeria, as part of the IVON trial [31]. The IVON trial was pre-registered in ClinicalTrials.gov on 26/07/2021, with Identifier No. NCT04976179. It is a randomized clinical trial evaluating the effectiveness and safety of intravenous iron versus oral ferrous sulphate for treatment of iron deficiency anaemia during pregnancy. The two most populous states in Nigeria, Lagos and Kano [32] were purposively selected for the trial to capture differentials in provision of health care like disparity in health systems processes, distribution of healthcare workers’ numbers, cadre and skills, and patient characteristics.

### Participants

**Fig. 1:**
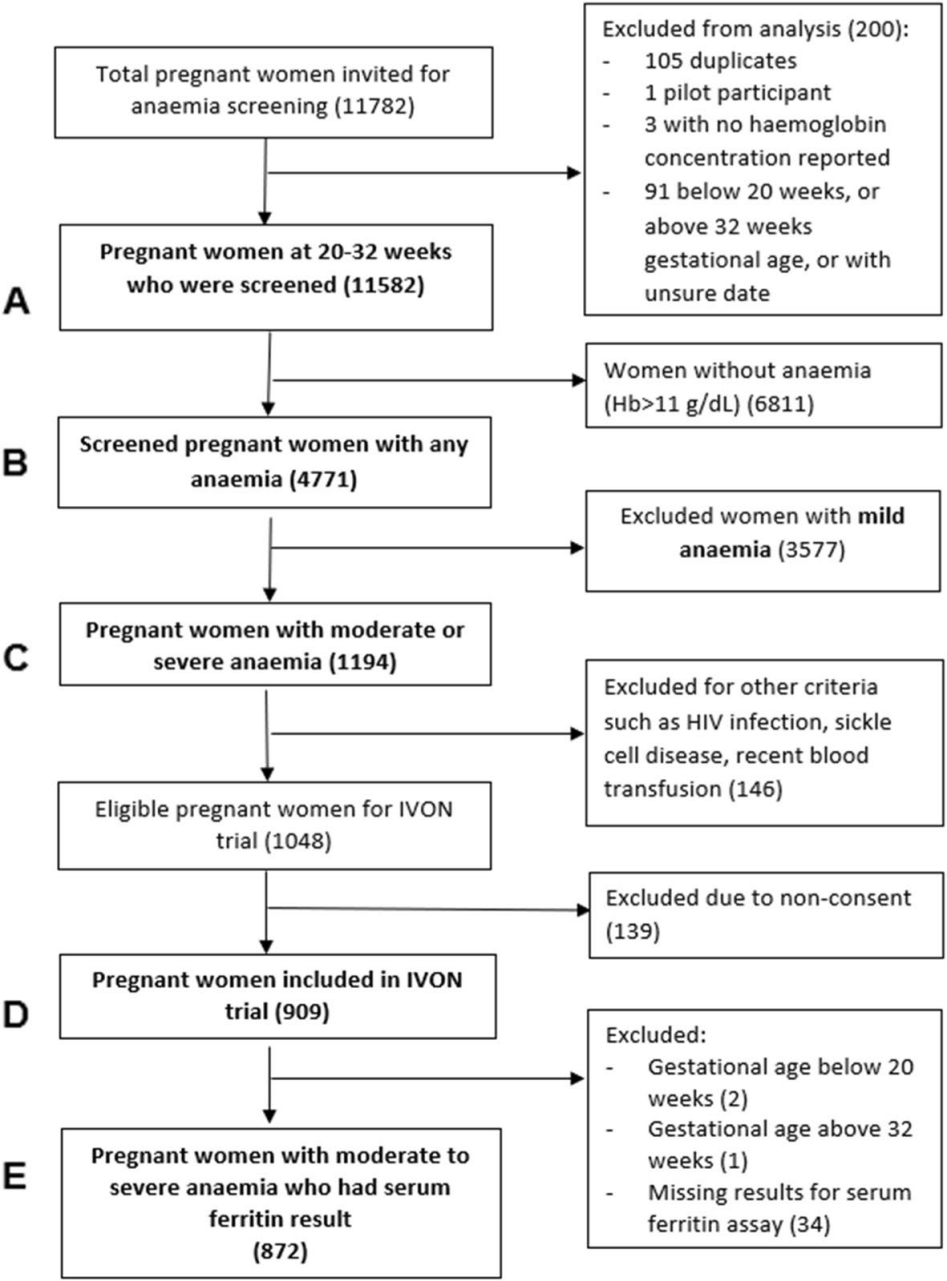
Flow chart showing inclusion of study participants (pregnant women at 20 – 32 weeks gestation) **A** Total number of pregnant women 20-32 weeks of gestation screened for anaemia **B** Number of pregnant women who are anaemic **C** Number of the pregnant women who had moderate or severe anaemia **D** Number of women enrolled into IVON trial **E** Number of IVON participants with serum ferritin result at enrolment, included in this study The IVON trial is a randomized controlled clinical trial evaluating the effectiveness and safety of oral ferrous sulphate versus intravenous ferric carboxymaltose for treatment of iron deficiency anaemia in pregnant Nigerian women

Participants included in this study were 872 pregnant women aged 15-49 years and at 20-32 weeks gestational age enrolled into the IVON trial between August 9, 2021, and October 7, 2022. A sample size of 872 participants would be more than sufficient to estimate the prevalence of iron deficiency among pregnant women with anaemia, based on a prevalence of 63.6% from previous study in a similar setting, using Cochran’s formula and adjusting for 20% attrition [33, 34]. Excluded from the IVON trial and this study were women with haemoglobinopathies, history of blood transfusion in the last three months, active bleeding from any part of the body, recent major surgery, autoimmune diseases, chronic kidney disease, cancer, human immunodeficiency virus infection (HIV), and clinically confirmed malabsorption syndrome. Figure 1 shows the inclusion flow chart for this study.

### Data collection

As part of routine ANC, all pregnant women attending the health facilities between 20 – 32 weeks gestational age had their haemoglobin (Hb) concentration determined during screening using Hemocue Hb 301 system. *Any anaemia* refers to anaemia of any severity, which in pregnancy is taken to be Hb concentration < 11 g/dL [35]. *Mild anaemia* in pregnancy: Hb concentration from 10 to < 11 g/dL [35]. *Moderate anaemia* in pregnancy: Hb concentration from 7 to < 10 g/dL [35]. *Severe anaemia* in pregnancy: Hb concentration < 7 g/dl [35]. We used a cut off value of 30 ng/mL for serum ferritin concentration in pregnant women to define ID in this study as it was found to have a higher sensitivity of 92% and a specificity of 98%, and correlates well with bone marrow iron stores, compared to a cut off of 12 ng/mL with sensitivity of 37.5% and specificity of 93.7% [36, 37]. The UK guideline also recommends treatment of pregnant women for iron deficiency when serum ferritin concentration is < 30 ng/mL [38]. At the time of this study, there was a lack of ANC guideline on IDA in Nigeria.

Women found to have moderate or severe anaemia (haemoglobin concentration below 10 g/dL) were invited into the IVON trial following informed consent. An interview was conducted with the consenting woman by a trained research nurse using an online questionnaire on Research Electronic Data Capture (REDCap) hosted by Queen Mary University of London [39]. Data in both the screening and enrolment databases were analysed for this study. Data collected during screening included the health facility name, and self-reported information such as maternal age, gestational age from the medical records and Hb level measured at the screening. Data collected during enrolment included self-reported information such as parity, marital status, ethnicity, place of residence, level of education and occupation of participant, male partner’s occupation (if applicable), and monthly income of participant. Food frequency assessment and participant’s weight and height measurements were collected at enrolment by the research nurse. All study participants were screened for malaria at enrolment using SD BIOLINE Malaria Ag P.f rapid diagnostic test kit [40]. Those who screened positive for malaria were treated with an artemisinin-based combination therapy and offered intermittent preventive therapy (IPT) subsequently to minimize the risk of recurrence, while those who screened negative for malaria were initiated on IPT if not already initiated.

### Main outcome

The binary outcome variable: IDA was defined as serum ferritin < 30 ng/mL. To measure serum ferritin, four millilitres (4 mL) of blood was collected from each participant, with Hb concentration < 10 g/dL, into serum separator tubes. The serum extracted was used for serum ferritin assay at Synlab Laboratories Limited, an internationally accredited laboratory, using ARCHITECT Ferritin assay method [41].

### Independent variables

Marital status was categorized as single, cohabiting, married, or divorced. Ethnicity was categorised as Igbo, Yoruba, Hausa, and others. Place of residence was categorised as urban or rural. Highest level of education was categorised as no formal education, some primary or completed primary, completed secondary, or completed tertiary or higher. Age was categorised into three groups: 15 – 19, 20 – 34, and ≥ 35 years. Body mass index was categorised as > 18.5 kg/m^2^ (underweight), 18.5 – < 25 kg/m^2^ (normal), 25 – < 30 kg/m^2^ (overweight), and ≥ 30 kg/m^2^ (obese). Parity was categorized as 0, 1, 2 – 4, and 5 and above, based on the number of pregnancies a woman has carried to 28 weeks gestational age and above. Late second trimester was defined as gestational age between 20 – 26 weeks, and early third trimester was defined as gestational age between 27 – 32 weeks.

Household socioeconomic status (SES) is a measure of an individual or family’s access to resources and it influences health-related outcomes and exposures [42]. It was defined using the Nigerian classification system by Ibadin et al. (2021) taking into consideration the participant and her male partner’s (excluded for the unmarried) occupation and level of education, and which generates scores of 1 – 6; 1 indicating the highest grade and 6 the lowest grade [43]. All participants who had a final score of 1 – 2 were categorised as being in upper socioeconomic class, final score of 3 – 4 as middle class, and 5 – 6 as lower socioeconomic class [43].

Dietary intake was assessed using a pre-tested 17-item food frequency questionnaire (FFQ), adapted from a previous tool [44]. It was redesigned for this study and comprised locally available food items some of which have previously been reported to be iron chelators such as turmeric and tea [45, 46] and performed well on pretesting with a scale reliability coefficient of 0.71. The FFQ contained 15 food items and two types of hot beverages. For the food items, participants were asked to select the frequency of consumption presented as a Likert scale as everyday (always), 3 to 4 times a week (often), every 2 or 3 weeks (sometimes), and don’t eat (never). Edible kaolin clay was included as a food item because it is commonly consumed by pregnant black African women [47] and has previously been reported to be associated with IDA [48]. For beverages, participants selected the average number of cups ingested per day categorised into four groups: 0, 1, 2, 3 or more.

### Data analysis

Prevalence of any anaemia in pregnancy was calculated as percentage of all women screened who had Hb < 11 g/dl (B/A in Fig. 1). Prevalence of IDA was estimated as the percentage of women with iron deficiency (serum ferritin < 30 ng/ml) among all women diagnosed with moderate or severe anaemia included in this study. Categorical variables are described as percentages with a 95% confidence interval (CI). Pearson’s Chi square test was used to evaluate the difference in the prevalence of IDA among the pregnant women with moderate or severe anaemia in the study sample, and to compare the distribution of categorical variables. Normality testing was conducted on continuous variables like maternal age, gestational age, haemoglobin concentration and serum ferritin concentration using Shapiro Wilks test to determine their distribution in the study population and to know the most appropriate statistical test to use for analysis. Wilcoxon rank sum test was used to describe and compare the medians of continuous variables (age and gestational age at inclusion in study). The predictive probabilities for IDA at various gestational ages was plotted by doing post estimation for predictive margins following logistic regression.

Frequency of consumption of various food items and hot beverages was presented using a heat map generated on Microsoft Excel using conditional formatting. Frequency of consumption of food items was re-categorized as “no consumption” when frequency is never eaten and as “consumption” for other options. Association between each food item and anaemia was determined using Chi square test. Association of IDA with independent variables such as age group, place of residence, socioeconomic status, parity, and dietary intake were analysed using binary logistic regression.

For socioeconomic status, upper and middle class were combined because there were very few participants (women with moderate or severe anaemia) in the upper social class. Purposeful selection was used for multivariable logistic regression analysis using a 3-step approach [49]. Bivariate logistic regression was done in step 1 and *p*-value threshold set at 0.25 for selection of variables for the model. In step 2, multivariable logistic regression was conducted with variables selected in step 1, entered in the model, and *p*-value threshold set at 0.05 for variables that were retained in the model. In step 3, all variables selected in step 2 were retained in the model. Sociodemographic variables which might have an impact on incidence of anaemia such as age, parity, socioeconomic status, geographical location, and gestation age were also included in the model in step 3. Subsequently variables dropped in steps 1 and 2 were included in sequence and any variable that caused a 10% change in the beta coefficient of any of the other variables in the model was retained.

Of the 872 participants included in this study, < 3% were missing any variable, and all analysis were done using complete cases. Statistical analysis was conducted using STATA version 16.0 statistical [50].

### Ethics

Approval was obtained from the research ethics committees of all teaching hospitals, ministry of health or health service commission, and primary health care board before commencement of the IVON trial (details in the section on declaration). Eligible participants were invited for anaemia screening. They were counselled on the need for routine screening for anaemia during pregnancy and verbal consent obtained. Those with moderate or severe anaemia were further informed about IVON trial and invited to participate. They were informed that participation was voluntary and that they could withdraw participation at any time. An informed consent was signed by each participant before enrolment into IVON trial. Pregnant women aged 15 to less than 18 were considered able to provide consent themselves based on the International Ethical Guidelines for Health-related Research Involving Humans by the World Health Organization in 2016 which stated that in certain circumstances, minors may be considered emancipated if they are living independently, are married, have children of their own, or are pregnant [51]. In addition, the Federal Ministry of Health, Nigeria, in a report in 2014 deemed adolescents aged 14 and above fit to provide consent themselves in non-surgical sexual and reproductive health research if they are married, head of a household or emancipated [52].

## Results

A total of 11,582 pregnant women were screened for anaemia, Fig. 1A including 5,182 women (44.7%) from Lagos and 6,400 (55.3%) from Kano state. A total of 3,681 (31.8%) of the women were screened in primary health centres, 6,213 (53.6%) in secondary health facilities, and 1,688 (14.6%) in tertiary facilities.

The prevalence of anaemia was 4,771/11,582 (41.2%, 95%CI: 40.3–42.1), and moderate or severe anaemia was 1,194/11,582 (10.3%, 95%CI: 9.8–10.9), as shown on Fig. 1B and C. Of the 1,194 pregnant women with moderate or severe anaemia, 1,048 were eligible for the IVON trial, and 909 (86.7%) of them consented Fig. 1D. Thirty-seven of the 909 participants were excluded from this study because one was enrolled after 32 weeks gestational age, two were enrolled below 20 weeks due to incorrect dating, and thirty-four did not have their serum ferritin reported, Fig. 1E*.*

A total of 872 pregnant women with moderate or severe anaemia were included in the analysis. The median age of the study participants was 27 (IQR: 23–32) years, with a median parity of 1 (IQR: 0–3), and 81.6% having at least secondary level of education, Table 1. Three hundred and fifty-nine had serum ferritin concentration < 30 ng/mL, meaning that iron deficiency was observed among 41.2% (95%CI: 37.9–44.5) of the women with moderate or severe anaemia (Table 2*)*. Among pregnant women with moderate or severe anaemia, serum ferritin concentration was significantly lower among women enrolled in the early third trimester compared to those enrolled in late second trimester (*p*-value < 0.001), Table 2. The prevalence of malaria parasitaemia was 5.9% (95%CI: 4.4–7.7).

**Table 1:** Characteristics of pregnant women with moderate or severe anaemia stratified by iron status (*n* = 872)

	**All participants** **(*N* = 872)**	**Iron deficient** **(*n* = 359)**	**Not iron deficient** **(*n* = 513)**	***p*-value**
**Median age** (IQR)** years**	27 (23 – 32)	27 (22 – 33)	28 (23 – 32)	0.868
**Median gestational age at enrolment** (IQR) (weeks)	25 (22 – 28)	26 (23 – 28)	24 (22 – 27)	< 0.001
**Median parity** (IQR)	1 (0—3)	1 (0—3)	1 (0—3)	0.206
**Place of residence** (*n* = 870) (%)				
Lagos				
Rural	43 (9.8)	16 (9.5)	27 (10.0)	0.762
Urban	394 (90.2)	152 (90.5)	242 (90.0)	
Kano				
Rural	27 (6.2)	14 (7.4)	13 (5.4)	0.389
Urban	406 (93.8)	176 (92.6)	230 (94.7)	
**Highest level of education** (*n* = 869) (%)				
No formal education	53 (6.1)	27 (7.5)	26 (5.1)	0.084
Some or completed primary	107 (12.3)	40 (11.2)	67 (13.1)	
Completed secondary	447 (51.4)	196 (54.8)	251 (49.1)	
Completed tertiary	262 (30.2)	95 (26.5)	167 (32.7)	
**Socioeconomic status** (*n* = 872) (%)				
Upper	18 (2.1)	6 (1.7)	12 (2.3)	0.470
Middle	400 (45.9)	158 (44.0)	242 (47.2)	
Lower	454 (52.0)	195 (54.3)	259 (50.5)	
**Income** (*n* = 869) (%)				
Less than ₦20,000	173 (19.9)	78 (21.8)	95 (18.6)	0.141
₦21,000 – ₦50,000	317 (36.5)	140 (39.1)	177 (34.6)	
₦51,000 – ₦100,000	150 (17.3)	63 (17.6)	87 (17.0)	
Greater than ₦100,000	76 (8.8)	28 (7.8)	48 (9.4)	
Not applicable/ no salary	106 (12.2)	33 (9.2)	73 (14.3)	
Undisclosed	47 (5.4)	16 (4.5)	31 (6.1)	
**Body mass index** (*n* = 871) (%)				
Underweight (< 18.5 kg/m^2^)	49 (5.6)	18 (5.0)	31 (6.0)	0.272
Normal (18.5—< 25.0 kg/m^2^)	472 (54.2)	205 (57.3)	267 (52.1)	
Overweight (25.0—< 30.0 kg/m^2^)	235 (27.0)	96 (26.8)	139 (27.1)	
Obese (> 30.0 kg/m^2^)	115 (13.2)	39 (10.9)	76 (14.8)	

₦ Nigerian naira. The *p*-values for continuous variables were obtained from Wilcoxon rank-sum test, and for categorical variables from Chi square test

**Table 2:** Haemoglobin and serum ferritin concentration in study participants presented by gestational age at enrolment (*n* = 872)

	**All participants, *n* = 872**	**Enrolled in late second trimester** **(20 – 26 weeks)**, ***n* = 561**	**Enrolled in early third trimester** (27 – 32 weeks), *n* = 311	***p*-value**
**Median haemoglobin concentration (IQR), g/dL**	9.3 (8.9 – 9.7)	9.4 (8.9 – 9.7)	9.3 (8.8–9.7)	0.248^a^
**Median serum ferritin concentration (IQR), ng/mL**	38.4 (18.3 – 74.2)	44.9 (21.7 – 83.5)	29.8 (15.4 – 62.4)	< 0.001^a^
**Serum ferritin category, frequency (%)**				
Very low < 15 ng/mL	164 (18.8)	88 (15.7)	76 (24.4)	0.001^b^
Low 15 to < 30 ng/mL	195 (22.4)	115 (20.5)	80 (25.7)	
Normal 30 – < 200 ng/mL	454 (52.1)	318 (56.7)	136 (43.7)	
High ≥ 200 ng/mL	59 (6.8)	40 (7.1)	19 (6.1)	
**Prevalence of IDA at serum ferritin concentration cut off < 30 ng/mL (95%CI)**	41.2 (37.9 – 44.5)	36.2 (32.2 – 40.3)	50.2 (44.5 – 55.9)	< 0.001^b^

*IQR* Interquartile range, *95%CI* 95% Confidence Interval

^a^Wilcoxon rank-sum test

^b^Chi square test

**Fig. 2:**
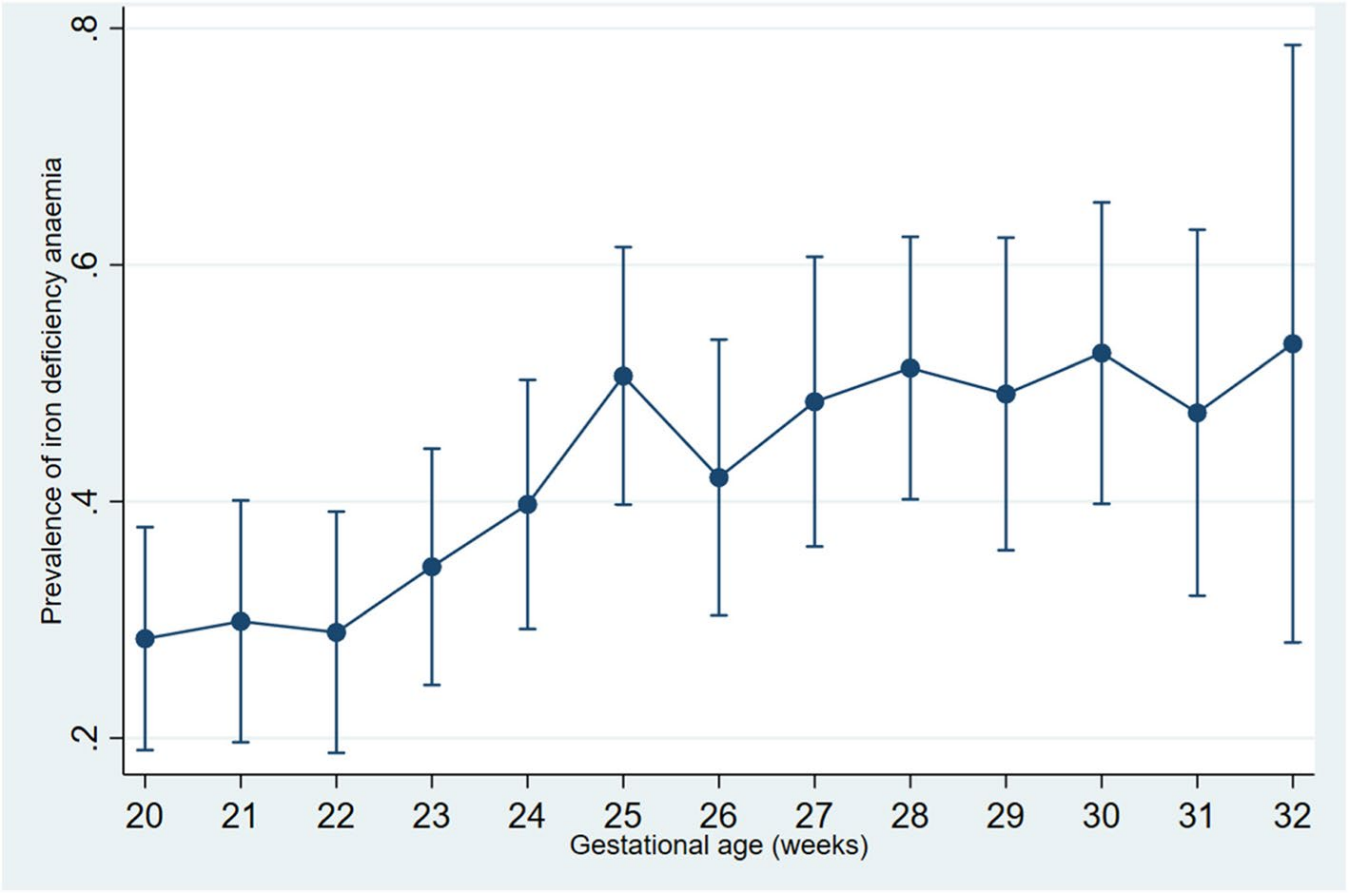
Prevalence of iron deficiency anaemia among pregnant women with moderate or severe anaemia, by gestational age in weeks (*n* = 872) Pearson chi2(12) = 30.1859 Pr = 0.003

Among the 872 women with moderate or severe anaemia, sociodemographic and anthropometric characteristics such as age, parity, place of residence, level of education, socioeconomic status, income, and body mass index, among those with and without IDA were similar (Table 2*)*. However, contribution of iron deficiency increased with gestational age in pregnant women with moderate or severe anaemia, from 28.4% (95%CI: 19.3–39.0%) at 20 weeks to 53.3% (95%CI: 26.6 – 78.7%) at 32 weeks, (Fig. 2).

### Food consumption

**Fig. 3:**
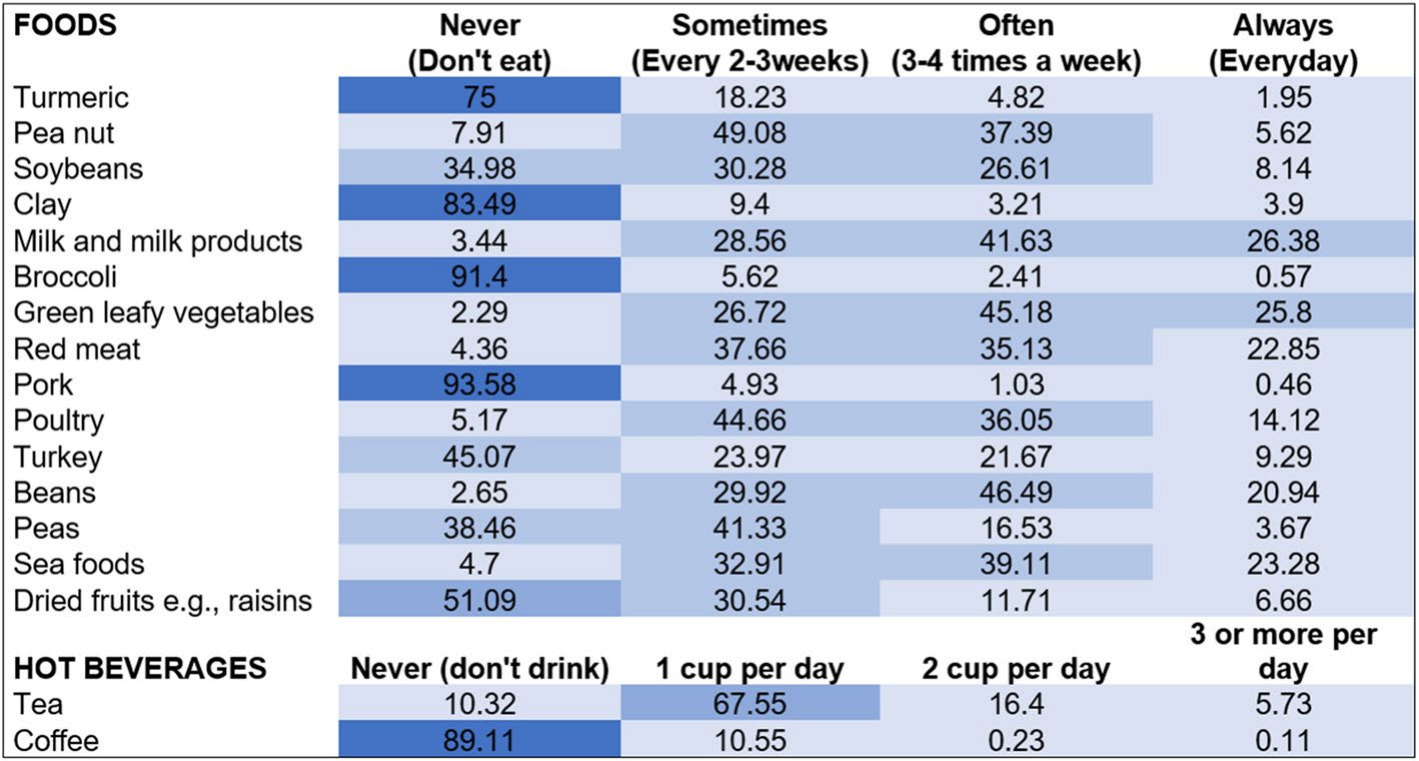
Heat map showing frequencies of food and beverage intake among study participants (*n* = 872) Figures are presented as percentage of study participants who consume food item at specified frequency.  75% or greater,  50-74.9%,  25-49.9%,  Less than 25%

Among the items in the FFQ, participants commonly consumed food items such as peanuts, soybeans, milk and milk products, green vegetables, red meat, poultry, beans, sea food, and beverages such as tea; most at a consumption frequency of every 2-3 weeks or 3–4 times a week (Fig. 3).

The prevalence of edible kaolin clay consumption among pregnant women with moderate or severe anaemia was 16.5% (95%CI: 14.1–19.2). The prevalence was found to be significantly higher in Kano state (19.4%, 95%CI: 15.8 – 23.4) compared to Lagos state (13.7%, 95%CI: 10.6 – 17.2%), *p* = 0.023. Compared to participants from Lagos, a significantly higher percentage of women in Kano state consumed turmeric (21.9% vs. 28.2%, *p* = 0.033) and soybeans (57.8% vs. 72.5%, *p* < 0.001). Compared to Kano, significantly more pregnant women in Lagos consumed turkey (45.5% vs. 64.2%, *p* < 0.001) and coffee (8.6% vs. 13.0%, *p* < 0.001). The consumption patterns of other foods were similar across the two states (*p* > 0.05).

Table 3 shows the results of bivariate association between food consumption by women with moderate or severe anaemia and IDA. There was a statistically significant association between any consumption of edible kaolin clay, soybeans, and green leafy vegetables and iron deficiency among pregnant women with moderate or severe anaemia. A larger proportion of those who consume soybeans had IDA compared to those who do not (41.6% versus 36.2%, *p* < 0.001). A larger proportion of those who consume edible kaolin clay had IDA compared to those who do not (54.2% versus 38.6%, *p* = 0.001). Whereas a greater proportion of pregnant women who do not consume green leafy vegetables had IDA compared to those who do (65% versus 41%, *p* = 0.029).

**Table 3:** Association between consumption frequency and iron deficiency in pregnant women with moderate or severe anaemia

Food/beverage	Iron deficient (*n* = 359)	Not iron deficient (*n* = 513)	*p*-value
Turmeric			
No consumption (*n* = 654)	261 (39.9)	393 (60.1)	0.190
Consumption (*n* = 218)	98 (45.0)	120 (55.0)	
Pea nuts			
No consumption (*n* = 69)	25 (36.2)	44 (63.8)	0.385
Consumption (*n* = 803)	334 (41.6)	469 (58.4)	
**Soybeans**			
No consumption (*n* = 305)	100 (32.8)	205 (67.2)	< 0.001
Consumption (*n* = 567)	259 (45.7)	308 (54.3)	
**Edible kaolin clay**			
No consumption (*n* = 728)	281 (38.6)	447 (61.4)	0.001
Consumption (*n* = 144)	78 (54.2)	66 (45.8)	
Milk and milk products			
No consumption (*n* = 30)	14 (46.7)	16 (53.3)	0.534
Consumption (*n* = 842)	345 (41.0)	497 (59.0)	
Broccoli			
No consumption (*n* = 796)	325 (40.8)	471 (59.2)	0.449
Consumption (*n* = 75)	34 (45.3)	41 (54.7)	
**Green leafy vegetables**			
No consumption (*n* = 20)	13 (65.0)	7 (35.0)	0.029
Consumption (*n* = 851)	346 (40.6)	505 (59.3)	
Red meat			
No consumption (*n* = 38)	19 (50.0)	19 (50.0)	0.261
Consumption (*n* = 833)	340 (40.8)	493 (59.2)	
Pork			
No consumption (*n* = 815)	339 (41.6)	476 (58.4)	0.387
Consumption (*n* = 56)	20 (35.7)	36 (64.3)	
Poultry such as chicken and eggs			
No consumption (*n* = 45)	14 (31.1)	31 (68.9)	0.155
Consumption (*n* = 825)	345 (41.8)	480 (58.2)	
Turkey			
No consumption (*n* = 393)	171 (43.5)	222 (56.5)	0.212
Consumption (*n* = 478)	188 (39.3)	290 (60.7)	
Beans			
No consumption (*n* = 23)	10 (43.5)	13 (56.5)	0.825
Consumption (*n* = 845)	348 (41.2)	497 (58.8)	
Peas			
No consumption (*n* = 334)	138 (41.3)	196 (58.7)	0.980
Consumption (*n* = 536)	221 (41.2)	315 (58.8)	
Sea food			
No consumption (*n* = 41)	14 (34.1)	27 (65.9)	
Consumption (*n* = 830)	345 (41.6)	485 (58.4)	
Dried fruits e.g., raisins			
No consumption (*n* = 444)	186 (41.9)	258 (58.1)	0.650
Consumption (*n* = 426)	172 (40.4)	254 (59.6)	
Tea			
No consumption (*n* = 90)	36 (40.0)	54 (60.0)	0.804
Consumes (*n* = 781)	323 (41.3)	458 (58.6)	
Coffee			
No consumption (*n* = 777)	322 (41.4)	455 (58.6)	0.699
Consumes (*n* = 94)	37 (39.4)	57 (60.6)	

Figures presented as frequency (percentage of row total). Hypothesis testing done with Chi square test. Missing values – 1 for poultry, 3 for beans, 1 for peas, and 1 for dried fruits

Tables 4, 5, 6 and 7 show the results of crude and multivariable logistic regression analysis of factors associated with IDA among pregnant women with moderate or severe anaemia. The adjusted odds for IDA was 57% higher in those at 27-32 weeks gestation compared to those at 20-26 weeks (95%CI:1,15-2.15). The adjusted odds for IDA was 70% higher in pregnant women aged 15-19 with moderate or severe anaemia compared to those in age group 20-34 years, though not statistically significant (aOR:1.70, 95%CI:0.91-3.18).

**Table 4:** Sociodemographic predictors of iron deficiency in pregnant women with moderate or severe anaemia

**Predictors**	**Bivariate analysis**			**Multivariable analysis**^a^	
	**OR (95%CI)**	***p*-value**	**LR *p*-value**	**aOR (95%CI)**	***p*-value**
**Age (years)**					
15 – 19	1.59 (0.89 – 2.84)	0.119	0.154	1.70 (0.91 – 3.18)	0.098
20 – 34	1.00			1.00	
35 years and above	1.28 (0.48 – 1.56)	0.189		1.19 (0.79 – 1.79)	0.396
**Parity**					
0	1.00		0.752	1.00	
1	1.10 (0.76 – 1.60)	0.618		1.15 (0.76 – 1.72)	0.505
2 – 4	1.21 (0.83 – 1.77)	0.331		1.14 (0.74 – 1.74)	0.552
5 and above	1.16 (0.82 – 1.66)	0.405		1.07 (0.72 – 1.59)	0.739
**Location of residence**					
Lagos	1.00	-	0.112	1.00	
Kano	1.24 (0.95 – 1.63)	0.113		0.99 (0.71 – 1.40)	0.974
**Socioeconomic class**					
Middle/ upper	1.00	-	0.253	1.00	-
Lower	0.85 (0.65 – 1.12)	0.254		1.00 (0.71 – 1.41)	0.986
**Gestational age**					
Late second trimester (20–26 weeks)	1.00	-	< 0.001*	1.00	-
Early third trimester (27–32 weeks)	1.77 (1.34 – 2.34)	** < 0.001**		1.57 (1.15 – 2.15)	**0.004**
**Body mass index**					
Underweight (< 18.5 kg/m^2^)	0.75 (0.41 – 1.38)	0.362	0.257	-	
Normal weight (≥ 18.5—< 25.0 kg/m^2^)	1.00			-	
Overweight (≥ 25.0—< 30 kg/m^2^)	0.90 (0.65 – 1.23)	0.499		-	
Obese (≥ 30 kg/m^2^)	0.65 (0.43 – 1.02)	0.062		-	
**Malaria**					
Absent	1.00		< 0.001*	1.00	-
Present	0.18 (0.08 – 0.43)	** < 0.001**		0.15 (0.06 – 0.38)	** < 0.001**

*OR* crude odds ratio, *aOR* adjusted odds ratio, *95%CI* 95% confidence interval, *LR* likelihood ratio

Total number of participants = 872. For the multivariable model, likelihood ratio (LR) *p*-value < 0.001, and Hosmer and Lemeshow goodness of fit *p*-value = 0.274

^a^Logistic regression analysis adjusts for age, parity, residence, socioeconomic status, gestational age, malaria parasitaemia and foods entered into the multivariable model as shown in Tables 4, 5 and 6

**Table 5:** Dietary predictors of iron deficiency in pregnant women with moderate or severe anaemia

Predictors	Bivariate analysis			Multivariable analysis^a^	
	**OR (95%CI)**	***p*-value**	**LR *p*-value**	**aOR (95%CI)**	***p*-value**
**Edible kaolin clay**					
Never—Don’t eat	1.00	-	< 0.001*	1.00	-
Sometimes – every 2–3 weeks	1.07 (0.67 – 1.70)	0.779		0.89 (0.54 – 1.47)	0.647
Often – 3–4 times a week	2.11 (0.99 – 4.54)	0.054		1.56 (0.70 – 3.48)	0.281
Always – everyday	9.21 (3.52 – 24.06)	** < 0.001**		9.13 (3.27 – 25.48)	** < 0.001**
**Milk**					
Never—Don’t eat	1.00	-	0.688	1.00	-
Sometimes – every 2–3 weeks	0.77 (0.36 – 1.64)	0.494		1.01 (0.41 – 2.44)	0.989
Often – 3–4 times a week	0.75 (0.36 – 1.59)	0.461		0.97 (0.40 – 2.34)	0.941
Always – everyday	0.89 (0.42 – 1.92)	0.775		1.13 (0.46 – 2.81)	0.787
**Green leafy vegetables**					
Never—Don’t eat	1.00	-	0.194	1.00	-
Sometimes – every 2–3 weeks	0.36 (0.14 – 0.95)	**0.038**		0.36 (0.13 – 0.98)	**0.046**
Often – 3–4 times a week	0.37 (0.14 – 0.95)	**0.038**		0.34 (0.13 – 0.93)	**0.036**
Always – everyday	0.37 (0.14 – 0.97)	**0.043**		0.26 (0.09 – 0.73)	**0.011**
**Red meat**					
Never—Don’t eat	1.00	-	0.253	1.00	-
Sometimes – every 2–3 weeks	0.77 (0.39 – 1.51)	0.453		0.71 (0.33 – 1.53)	0.386
Often – 3–4 times a week	0.69 (0.35 – 1.36)	0.283		0.70 (0.33 – 1.48)	0.349
Always – everyday	0.57 (0.28 – 1.14)	0.111		0.59 (0.27 – 1.29)	0.186
**Soybeans**					
Never—Don’t eat	1.00	-	0.001*	1.00	-
Sometimes – every 2–3 weeks	1.53 (1.08 – 2.15)	**0.015**		1.49 (0.98 – 2.26)	0.060
Often – 3–4 times a week	1.97 (1.39 – 2.80)	** < 0.001**		1.78 (1.12 – 2.82)	**0.015**
Always – everyday	1.67 (0.99 – 2.83)	0.055		1.45 (0.75 – 2.82)	0.270
**Turkey**					
Never—Don’t eat	1.00	-	0.108	1.00	-
Sometimes – every 2–3 weeks	0.96 (0.69 – 1.35)	0.827		1.40 (0.93 – 2.10)	0.107
Often – 3–4 times a week	0.65 (0.46 – 0.94)	0.022*		0.90 (0.57 – 1.43)	0.669
Always – everyday	1.04 (0.64 – 1.68)	0.877		1.45 (0.78 – 2.68)	0.242

*OR* crude odds ratio, *aOR* adjusted odds ratio, *95%CI* 95% confidence interval, *LR* likelihood ratio

Total number of participants = 872. For the multivariable model, likelihood ratio (LR)* p*-value < 0.001, and Hosmer and Lemeshow goodness of fit *p*-value = 0.274

^a^Logistic regression analysis adjusts for age, parity, residence, socioeconomic status, gestational age, malaria parasitaemia and foods entered into the multivariable model as shown in Tables 4, 5 and 6

**Table 6:** Dietary predictors of iron deficiency in pregnant women with moderate or severe anaemia

Predictors	Bivariate analysis			Multivariable analysis^a^	
	**OR (95%CI)**	***p*-value**	**LR *p*-value**	**aOR (95%CI)**	***p*-value**
**Peanuts**					
Never – Don’t eat	1.00	-	0.199	1.00	-
Sometimes – every 2–3 weeks	1.14 (0.67 – 1.93)	0.633		1.07 (0.60 – 1.92)	0.808
Often – 3–4 times a week	1.33 (0.78 – 2.28)	0.296		1.19 (0.64 – 2.19)	0.583
Always – everyday	1.99 (0.94 – 4.19)	0.949		2.10 (0.90 – 4.90)	0.085
**Tea**					
Never – Don’t drink	1.00	-	0.892	1.00	-
One cup per day	1.03 (0.65 – 1.62)	0.907		0.84 (0.50 – 1.40)	0.502
Two cups per day	1.18 (0.69 – 2.02)	0.542		0.98 (0.52 – 1.82)	0.943
Three or more cups per day	1.09 (0.54 – 2.19)	0.817		0.91 (0.42 – 2.00)	0.820
**Broccoli**					
Never – Don’t eat	1.00	-	0.096	-	-
Sometimes – every 2–3 weeks	1.78 (1.00 – 3.18)	0.052		-	-
Often – 3–4 times a week	0.58 (0.22 – 1.51)	0.264		-	-
Always – everyday	0.36 (0.04 – 3.26)	0.365		-	-
**Pork**					
Never – Don’t eat	1.00	-	0.571	-	-
Sometimes – every 2–3 weeks	0.92 (0.49 – 1.72)	0.789		-	-
Often – 3–4 times a week	0.40 (0.08 – 1.94)	0.257		-	-
Always – everyday	0.47 (0.05 – 4.52)	0.512		-	-
**Poultry such as eggs, chicken**					
Never – Don’t eat	1.00	-	0.088	-	-
Sometimes – every 2–3 weeks	1.76 (0.91 – 3.40)	0.096		-	-
Often – 3–4 times a week	1.32 (0.67 – 2.57)	0.424		-	-
Always – everyday	1.88 (0.91 – 3.88)	0.088		-	-
**Turmeric**					
Never – Don’t eat	1.00	-	0.139	-	-
Sometimes – every 2–3 weeks	1.31 (0.92 – 1.85)	0.132		-	-
Often – 3–4 times a week	0.75 (0.39 – 1.45)	0.395		-	-
Always – everyday	2.15 (0.81 – 5.71)	0.126		-	-

*OR* crude odds ratio, *aOR* adjusted odds ratio, *95%CI* 95% confidence interval, *LR* likelihood ratio

Total number of participants = 872. For the multivariable model, likelihood ratio (LR) *p*-value < 0.001, and Hosmer and Lemeshow goodness of fit *p*-value = 0.274

^a^Logistic regression analysis adjusts for age, parity, residence, socioeconomic status, gestational age, malaria parasitaemia and foods entered into the multivariable model as shown in Tables 4, 5 and 6

**Table 7:** Dietary predictors of iron deficiency in pregnant women with moderate or severe anaemia

Predictors	Bivariate analysis			Multivariable analysis^a^	
	**OR (95%CI)**	***p*-value**	**LR *p*-value**	**aOR (95%CI)**	***p*-value**
**Beans**					
Never—Don’t eat	1.00		0.980	-	-
Sometimes – every 2–3 weeks	0.89 (0.37 – 2.10)	0.784		-	-
Often – 3–4 times a week	0.93 (0.40 – 2.18)	0.876		-	-
Always – everyday	0.89 (0.37 – 2.14)	0.796		-	-
**Peas**					
Never—Don’t eat	1.00		0.301	-	-
Sometimes – every 2–3 weeks	0.89 (0.66 – 1.21)	0.467		-	-
Often – 3–4 times a week	1.31 (0.88 – 1.94)	0.182		-	-
Always – everyday	0.97 (0.46 – 2.03)	0.939		-	-
**Sea foods**					
Never—Don’t eat	1.00		0.162	-	-
Sometimes – every 2–3 weeks	1.55 (0.78 – 3.08)	0.209		-	-
Often – 3–4 times a week	1.15 (0.58 – 2.27)	0.688		-	-
Always – everyday	1.54 (0.76 – 3.10)	0.231		-	-
**Dried fruits e.g., raisins**					
Never—Don’t eat	1.00	-	0.265	-	-
Sometimes – every 2–3 weeks	1.02 (0.75 – 1.39)	0.878		-	-
Often – 3–4 times a week	0.66 (0.42 – 1.05)	0.078		-	-
Always – everyday	1.13 (0.65 – 1.95)	0.670		-	-
**Coffee**					
Never—Don’t drink	1.00	-	0.913	-	-
One cup per day	0.92 (0.59 – 1.44)	0.730		-	-
Two cups per day	1.41 (0.09 – 22.67)	0.807		-	-
Three or more cups per day	1.00 (0.43 – 1.02)	-		-	-

*OR* crude odds ratio, *aOR* adjusted odds ratio, *95%CI* 95% confidence interval, *LR* likelihood ratio

Total number of participants = 872. For the multivariable model, likelihood ratio (LR) *p*-value < 0.001, and Hosmer and Lemeshow goodness of fit *p*-value = 0.274

Pregnant women with malaria parasitaemia had 85% lower odds for IDA compared to pregnant women without malaria parasitaemia (aOR:0.15, 95%CI:0.06–0.38). Consumption of green leafy vegetables was found to be associated with 64 – 74% lower adjusted odds of having IDA compared to those who did not consume green vegetables. Pregnant women who consumed edible kaolin clay every day had nine times higher adjusted odds of having IDA compared to those who did not consume it at all (*p* < 0.001).

## Discussion

This study showed that 10.3% of pregnant women recruited for the IVON trial at 20-32 weeks gestational age in public health facilities in Lagos and Kano states had moderate or severe anaemia, and 41.2% of those with moderate or severe anaemia were iron deficient. With increasing gestational age, the contribution of iron deficiency as a cause of anaemia increased from 1 in 4 pregnant women with moderate or severe anaemia at 20 weeks gestational age to 1 in 2 by 32 weeks. Dietary factors associated with higher odds of IDA included consumption of soybeans and edible kaolin clay, while consumption of green leafy vegetables reduced the odds.

The proportion of moderately-severely anaemic pregnant women with iron deficiency found in our study is consistent with an earlier systematic review showing that iron deficiency complicates 25 – 46% of anaemic pregnant women in Nigeria [26]. However, it is lower than prevalence of 64% in pregnant women with moderate or severe anaemia reported in a study in Zaria, a state in Northern Nigeria just like Kano [33]. The lower prevalence of IDA in our study may be partly explained by the differences in the exclusion criteria applied in both studies. We excluded pregnant women who were below 20 weeks or above 32 weeks gestational age, or had HIV, allergic to iron-containing medications or have autoimmune diseases and included only those who consented to partake in a clinical trial. Unlike the Zaria study, our study covered wider and different geographical areas, and recruited participants from all the three levels of public healthcare.

Our analysis confirmed published evidence that with increasing gestation, the incidence of IDA increases [53, 54]. Physiological changes in pregnancy such as an increase in plasma volume cause physiologic haemodilution, a plausible explanation for the increased incidence of anaemia during pregnancy [55]. However, this mechanism might not offer a plausible explanation for the increased incidence of IDA during pregnancy. Considering that pregnancy has been found to cause a two- to three-fold increase in iron demand for haematopoeisis and for the foetus [55], a possibility exists that the demand for iron during pregnancy increases as the foetus grows. However, whether the observed association between IDA and gestational age is a result of the anaemia, or the iron deficiency, or both is a question that calls for future research.

Although difference in prevalence of IDA across geographical locations might suggest location as a possible determinant for IDA, an association between state of residence and the prevalence of IDA was not found in our study. However, we found some disparities in dietary habit across states, as a higher proportion of pregnant women with moderate or severe anaemia in Kano compared to Lagos state consumed foods like soybeans and edible kaolin clay, which have been associated with IDA in this study. We found that daily consumption of edible kaolin clay was associated with a nine-fold increase in odds for IDA in pregnant women with moderate or severe anaemia in this study. Our study reported a prevalence of edible kaolin clay consumption of 16.5% in pregnant women with moderate and severe anaemia. The consumption of edible kaolin clay, during pregnancy, is well known among people from diverse cultures with prevalence of 35.4% in Cameroon [56], and 47.0 - 47.5% in Ghana [57, 58].

Some pregnant women eat edible kaolin clay to suppress pregnancy symptoms such as water brash and nausea, or just for the craving - pica [59, 60]. Edible kaolin clay is obtained from earth and contains kaolinite which prevents proper absorption of iron from the duodenum and might thus cause iron deficiency if consumed frequently [48]. Edible kaolin clay has been found to contain chemicals and heavy metals such as arsenic, lead, nickel, silicon, mercury, cadmium, anthraquinone, small amount of androgens and steroids, and some micro-organisms [61–63]. It will be beneficial to explore further the association between edible kaolin clay and IDA to determine if the association is a cause or an effect of IDA as earlier studies have given conflicting reports [48, 64].

Interestingly, eating soybeans 3 – 4 times a day was found to increase odds for IDA by 78% in this study. This conflicts earlier findings that soybeans reduces the incidence of anaemia and iron deficiency [65] because they contain low amounts of ferritin and thus enhance iron bioavailability [65–67]. Our finding is corroborated by a study in Chinese population which found a positive association between tofu (contains dried soybeans) intake and haemoglobin concentration, but an inverse association between tofu intake and serum ferritin levels in women [68]. The reason for this is unclear. However, it might be related to micronutrient damage from cooking methods [69] or due to differences in species of soybeans consumed in various regions as soybeans high in phenolic acids have been found to have a higher iron bioavailability compared to species low in phenolic acids [70]. There is a need to explore these possibilities in future research.

Green leafy vegetable consumption was found to be associated with a lower odds of IDA in this study. A recent randomized clinical trial conducted on children aged 4 – 9 years in Ghana involving nutritional intervention using green leafy vegetable powder found that children who consumed the powder had significantly lower prevalence of anaemia and vitamin A deficiency [71]. Similar association was found in a recent cross-sectional study on small scale female farmers in Tanzania [72]. Common green leafy vegetables in Nigeria such as Telfairia occidentalis popularly known as “Ugwu” and Amaranthus hybridus known locally as “Tete adayeba” have high iron concentrations of 0.70 ppm and 0.67 ppm respectively [73]. The high vitamin C content of green leafy vegetables facilitates the absorption and bioavailability of iron. Some studies found that in women with IDA, serum hepcidin and serum ferritin concentration were positively correlated with the frequency of intake of green leafy vegetables [74, 75].

Malaria parasitaemia was associated with lower odds of IDA in our study. Nevertheless, malaria parasitaemia is known to increase odds for anaemia by causing haemolysis [76]. Unlike anaemia which pathogenesis in relation to malaria parasitaemia is clear, the pathogenesis for iron deficiency is unclear. However, scholars propose that ferroportin, a transport protein in erythrocytes, prevents iron accumulation in erythrocytes [77]. This affects erythrocytic schizogony as malaria parasites cannot thrive in iron depleted red blood cells [78]. In addition, ferroportin has been found in abundance in erythrocytes in iron deficient states but diminished in iron replete states [77].

### Strengths and limitations

Our study has several strengths compared to previous work in Nigeria, including a large sample size of women screened for anaemia and inclusion of several health facilities at various levels of healthcare (primary, secondary, and tertiary) located in different parts of the country (Northern and Southern Nigeria) [33, 79]. However, the exclusion of pregnant women seeking ANC in private health facilities, restriction to clinical trial participants, and the exclusion of the population of pregnant women within the community who are not attending any antenatal clinic, limits the generalizability of our findings and might have introduced some bias. Our study participants comprised mostly urban women with higher education, than what we would expect in Nigeria as a whole, based on the NDHS data [25]. Of the pregnant women who received ANC in sub-Saharan Africa, 81.2% received care from the public sector while about 15% received ANC from the commercial and not-for-profit private health facilities [80]. We can also not completely rule out the possibility of residual confounding and reverse causality in this study, especially regarding the association with edible kaolin clay consumption.

Based on our findings, there is a need for advocacy on early registration for ANC and regular ANC appointments to facilitate prevention or early diagnosis of IDA bearing in mind the association with increasing gestational age. Screening for iron deficiency in anaemic pregnant women with risk factors for IDA should be considered globally especially in sub-Saharan Africa where the burden of IDA is highest. Focused dietary intervention programmes need to be included in guidelines for management of IDA especially in settings where the compliance rate for the use of haematinics during pregnancy is low. There is a need to discourage consumption of edible kaolin clay, as it is clear from this and other studies that frequent consumption of edible kaolin clay in large amount poses significant health risk; this can be integrated into health education sessions within ANC. Whether non-consumption of edible kaolin clay will reduce incidence of IDA during pregnancy remains debatable, as there has been arguments on possibility of edible kaolin clay consumption being an effect rather than a cause of IDA [48, 64].

Almost 60% of the moderately or severely anaemic pregnant women in this study did not have iron deficiency, suggesting that their anaemia is due to other factors. The prevalence of malaria parasitaemia was also low (5.9%), despite the enrolment period spanning through the peak period of malaria transmission, April to October, in Nigeria [81]. This raises the need to explore in future research other causes of anaemia in pregnant Nigerian women. It will also be of interest in future research to estimate the proportion of non-anaemic pregnant women who are iron-deficient as such women could be effectively managed with dietary modifications alone, considering that non-anaemic iron deficiency has been associated with a higher risk of developing anaemia in pregnancy, increased incidence of low birth weight and neurodevelopmental problems in infants of affected mother [82–84].

## Conclusion

In our study, 4 in 10 women with moderate or severe anaemia during pregnancy had IDA. We provide evidence for the protective effect of green leafy vegetables against IDA while self-reported consumption of edible kaolin clay and soybeans appeared to increase the odds of having IDA during pregnancy. Our findings have implications for practice and research. They call for evaluation of health education interventions and focused dietary advice and modification during pregnancy since these could potentially increase awareness and change behaviours that could reduce IDA among pregnant women with moderate or severe anaemia in Nigeria and beyond as strategies to improve maternal and perinatal outcomes. Our findings also raise other research questions worth exploring in future research to improve scientific knowledge worldwide on iron deficiency anaemia during pregnancy.

### Supplementary Information

**Additional file 1: Supplementary file 1.** Food frequency questionnaire

**Additional file 2: Supplementary file 2.** Summary of screening and inclusion into study by state and facility level.

## Data Availability

The enrolment data of the ongoing IVON trial was used for this study. The complete dataset analysed for this study is available in the Open Science Framework data repository platform. The link to the dataset is: https://osf.io/6g8fw/.

## Abbreviations

95%CI: 95% Confidence interval
ANC: Antenatal care
ANOVA: Analysis of variance
FFQ: Food frequency questionnaire
g/dL: Grammes per decilitre
Hb: Haemoglobin
HIV: Human immunodeficiency virus
IDA: Iron deficiency anaemia
IVON: Intravenous versus oral iron for iron deficiency anaemia in pregnant Nigerian women
kg/m2: Kilogramme per square metre
mL: Millilitre
NDHS: Nigeria Demographic Health Survey
ng/mL: Nanograms per millilitre
NGN: Nigerian naira
NHREC: National Health Research Ethics Committee
OR: Odds ratio
ppm: Parts per million
REDCap: Research electronic data capture
RLUs: Relative light units
RR: Relative risk
WHO: World Health Organization
