# Supplementary material for: Prevalence of and risk factors for iron deficiency among pregnant women with moderate or severe anaemia in Nigeria: a cross-sectional study
Source: BMC Pregnancy Childbirth. 2024 Jan 5;24:39. doi: 10.1186/s12884-023-06169-1 (PMC10768359; doi:10.1186/s12884-023-06169-1)
Supplement: Supplementary file 1 — Additional file 1: Supplementary file 1. Food frequency questionnaire [file 12884_2023_6169_MOESM1_ESM.docx]

**Supplementary file 1: FOOD FREQUENCY QUESTIONNAIRE**

*Tick the most appropriate response that best describes the frequency of consumption of the various food items listed.*

| **Food frequency** | **Every day (ALWAYS)** | **3 - 4 times a week (OFTEN)** | **Every 2 or 3 weeks (SOMETIMES)** | **Don’t eat (NEVER)** |
| --- | --- | --- | --- | --- |
| How often do you eat foods containing the following items? | | | | |
| Turmeric | O | O | O | O |
| Groundnuts | O | O | O | O |
| Soybeans | O | O | O | O |
| Clay | O | O | O | O |
| Milk | O | O | O | O |
| Broccoli | O | O | O | O |
| Green vegetables | O | O | O | O |
| Red meat | O | O | O | O |
| Pork | O | O | O | O |
| Poultry e.g., chicken, turkey | O | O | O | O |
| Beans | O | O | O | O |
| Peas | O | O | O | O |
| Sea foods e.g., shrimps, fish | O | O | O | O |
| Dried fruits e.g., raisins | O | O | O | O |
| **Beverage frequency** | **3 or more** | **2** | **1** | **0** |
| How many cups of the following beverages do you drink per day? | | | | |
| Tea | O | O | O | O |
| Coffee | O | O | O | O |
