# Supplementary material for: Prevalence of and risk factors for iron deficiency among pregnant women with moderate or severe anaemia in Nigeria: a cross-sectional study
Source: BMC Pregnancy Childbirth. 2024 Jan 5;24:39. doi: 10.1186/s12884-023-06169-1 (PMC10768359; doi:10.1186/s12884-023-06169-1)
Supplement: Supplementary file 2 — Additional file 2: Supplementary file 2. Summary of screening and inclusion into study by state and facility level. [file 12884_2023_6169_MOESM2_ESM.docx]

**Supplementary file 2: Summary of screening and inclusion into study by state and facility level**

| **Study site** | **Women screened (%), n = 11,582** | **Women with moderate and severe anaemia included in study, n = 872** |
| --- | --- | --- |
| **States** |  |  |
| Lagos state | 5182 (44.7) | 439 (50.3) |
| Kano state | 6400 (55.3) | 433 (49.7) |
| **Level of healthcare** |  |  |
| Primary level (I) | 3681 (31.8) | 246 (28.2) |
| Secondary level (II) | 6213 (53.6) | 558 (64.0) |
| Tertiary level (III) | 1688 (14.6) | 68 (7.8) |
|  |  |  |

*Figures are presented as frequency and percentage of total in each column.*
